# Osteoid Osteoma in an Elderly Patient: Diagnostic Challenges

**DOI:** 10.1177/10668969241308229

**Published:** 2025-01-08

**Authors:** Shunsuke Koga, Wei Du, Cara A. Cipriano, Kumarasen Cooper

**Affiliations:** 1 Department of Pathology and Laboratory Medicine, 21798 Hospital of the University of Pennsylvania, Philadelphia, Pennsylvania, USA; 2 Department of Orthopaedic Surgery, 21798 Hospital of the University of Pennsylvania, Philadelphia, Pennsylvania, USA

**Keywords:** osteoid osteoma, elderly, pathology

## Abstract

Osteoid osteoma is a benign bone tumor commonly affecting young individuals, with a rare occurrence in older adults. It typically presents with night pain relieved by nonsteroidal anti-inflammatory drugs and is characterized radiographically by a small, radiolucent nidus surrounded by reactive sclerosis. We present a 70-year-old female patient with persistent right hip pain, initially diagnosed as arthritis, who underwent total hip arthroplasty. Preoperative imaging findings were inconclusive, showing edema and degenerative changes without a definitive lesion. Histopathological examination of the arthroplasty specimen revealed a nodule consistent with osteoid osteoma. This report highlights the diagnostic challenge of osteoid osteoma in elderly patients, where symptoms may be atypical and overlap with other conditions like osteoarthritis.

## Introduction

Osteoid osteoma accounts for 11%–14% of all benign bone tumors.^1^ The diaphysis and metaphysis of the long bones are the most common sites, with approximately 50% involving the femur or tibia.^1^ Osteoid osteoma typically occurs in individuals between the ages of 10 and 20 years. It is rare in children under 5 or individuals over 40,^2^ and may not be commonly considered in the differential diagnosis of bone lesions in older adults. The typical presentation includes intermittent pain that worsens at night and is relieved by nonsteroidal anti-inflammatory drugs (NSAIDs). Radiographically, it is identified by a small radiolucent nidus surrounded by a zone of reactive sclerosis. CT is the modality of choice for diagnosing osteoid osteoma, while MRI can show better bone marrow edema and periostitis.^3^ We report a 70-year-old patient with osteoid osteoma, where the diagnosis was inconclusive through radiological assessment but was finally confirmed by pathological evaluation of the surgical resection specimen. This observation highlights the importance of considering osteoid osteoma as a differential diagnosis, even in patients beyond the typical age and symptoms.

## Case Report

**Figure 1.**
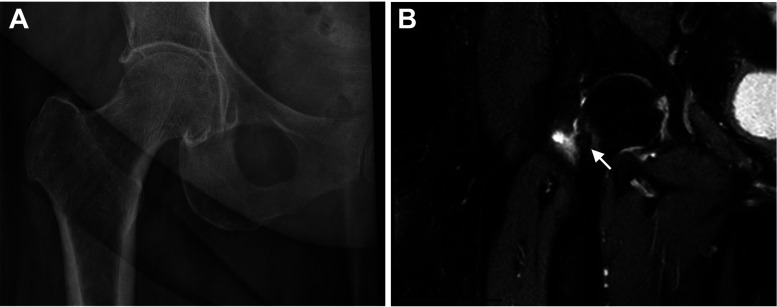
Representative radiological images. (A) An X-ray of the right hip shows moderate hip joint narrowing and degenerative joint disease, with no distinct lesion identified. (B) Coronal T2-weighted image of the right femur shows a 6.4mm round focus of intermediate signal intensity (arrow) contiguous with the cortical margin, along with surrounding bone marrow edema.

The patient was a 70-year-old woman with a history of bilateral knee replacements, occasional back pain with radicular symptoms, osteopenia, prediabetes, hypertension, and dyslipidemia who presented with a 1.5-year history of persistent right hip pain, initially diagnosed as arthritis. An X-ray performed at the outside hospital revealed mild to moderate bilateral hip joint narrowing, most pronounced on the right with severe medial joint space loss. Mild to moderate degenerative joint disease was also noted, with no distinct lesion identified (Figure 1A). An MRI from the same hospital showed mild to moderate heterogeneous edema in the mid-third and base of the right femoral neck and a 6.4 mm round focus of intermediate signal at the anterior margin of the femoral neck (Figure 1B), raising suspicion for osteoid osteoma.

When the same MR images were reviewed by radiologists at our institution, they disagreed with the prior description and attributed the findings to degenerative changes and osseous edema, with no evidence of a discrete lesion. The patient reported right groin and thigh pain, which worsened during prolonged standing and walking with limited range of motion. Her symptoms were partially relieved by ibuprofen. She denied specific night pain, fever, chills, weight changes, numbness, or tingling in the affected limb. Physical examination revealed an antalgic gait and pain with hip range of motion, particularly internal rotation. She had mild tenderness over the greater trochanter and anterior hip and pain with resisted hip flexion, but good overall strength. Given the persistent pain, limited response to conservative management, and the imaging findings—despite the discrepancy in interpretation—the patient opted for right total hip arthroplasty for both symptom relief and definitive diagnosis.

**Figure 2.**
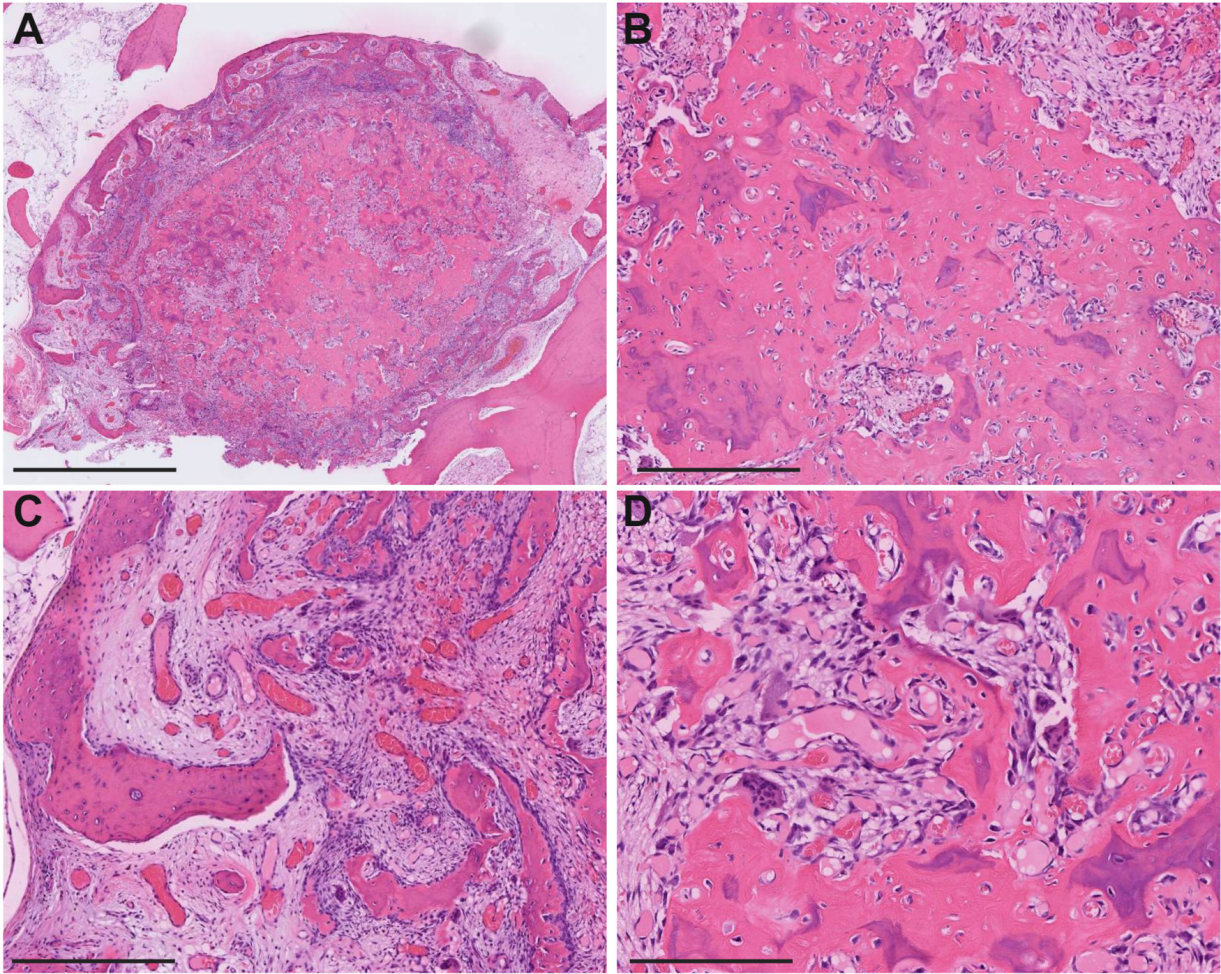
Representative histological images of the lesion. (A) Low magnification hematoxylin and eosin (H&E) stain of the resected specimen shows a well-defined lesion surrounded by sclerotic bone. (B–D) Higher magnification H&E stains reveal central sclerosis of the bone (B), peripheral formation of woven bone (C), vascular stroma (C), and evident osteoclastic and osteoblastic activity (D). Scale bars: A = 2mm; B–D = 400 μm.

During surgery, gross evidence of arthritis was noted. Examination of the resected femoral head revealed a gray, porous nodule measuring 0.8 × 0.7 × 0.1cm on the femoral shaft. Histopathological evaluation of this nodule showed central sclerosis with peripheral woven bone formation (Figure 2A to C). The stroma was vascular (Figure 2C), with evident osteoclastic and osteoblastic activity (Figure 2D). These pathological findings were consistent with osteoid osteoma. The patient recovered from surgery uneventfully, with complete resolution of symptoms postoperatively.

## Discussion

This report illustrates the challenges in diagnosing osteoid osteoma in elderly patients, partially because osteoid osteoma is exceedingly rare in this age group. A literature review of 95 patients revealed that only 4% of them were over 40 years old.^2^ In a case series of 43 patients with osteoid osteoma in older adults aged 35 years or older, only 5 patients (12%) were over 40, with the oldest being 66 years^4^ In a larger cohort, only 3 out of 102 patients (3%) were aged 70 years or older, and these older patients reported more severe pain compared to younger individuals.^5^ The oldest reported patient is a 77-year-old man with an osteoid osteoma in his hand, successfully treated with CT-guided percutaneous radiofrequency ablation.^6^ These studies emphasize that osteoid osteoma needs to be included in the differential diagnosis of painful sclerotic bone lesions in older adults to avoid delayed diagnosis and treatment.

The presentation of osteoid osteoma in older patients may be atypical or complicated by coexisting medical conditions. In this patient, classic symptoms such as night pain or relief with NSAIDs were absent. Rather, she had signs, symptoms, and imaging consistent with hip osteoarthritis, which likely contributed to her pain. It remains challenging the impact of her hip osteoarthritis from that of the osteoid osteoma, as the hip replacement addressed both conditions simultaneously.

The discrepancy in the radiological assessment of our patient may be due to atypical patient history and age, as well as complexity of the imaging characteristics. While MRI can reveal bone marrow edema and periostitis associated with osteoid osteoma, CT remains the modality of choice for accurately identifying and localizing the lesion, especially the central nidus, which is often obscured on radiographs and challenging to detect on MRI alone.^3^ Additionally, there may be fusiform cortical thickening and/or less reactive surrounding sclerosis in the older population.^3^ In our patient, the varied MRI interpretations highlight the importance of a comprehensive, multidisciplinary imaging review, ideally including CT scans, for accurate diagnosis.

The differential diagnosis for osteoid osteoma can be complicated due to overlapping clinical, radiographic, and histologic features.^7^ Osteoblastoma should be considered due to its similarity in histology; however, it typically presents as a larger lesion (>2 cm) and may demonstrate more aggressive growth without the reactive sclerosis.^8^ Aneurysmal bone cyst can also be considered, as it may present with giant cells and similar radiographic features; however, it is usually larger, expansile, and lacks the central nidus, showing instead multiple blood-filled cystic spaces.^9^ Fibrous dysplasia is another differential, usually presenting with bone pain and cortical expansion; however, it is lacking a central nidus and instead showing irregularly shaped trabeculae of woven bone within a fibrous stroma.^9^ Bone islands with reactive fibrous stroma might mimic osteoid osteoma due to the presence of reactive stroma, but they do not exhibit the intense osteoblastic activity or central nidus.^10^ Accurate histopathological evaluation is crucial to distinguishing these entities.

In conclusion, osteoid osteoma should be considered in the differential for a small cortical bone lesion, even in atypical patient populations. This case report contributes to the limited literature on osteoid osteoma in the elderly and illustrates the need for a comprehensive approach to patient evaluation and management.
